# Distinct contributions of cathelin‐related antimicrobial peptide (CRAMP) derived from epithelial cells and macrophages to colon mucosal homeostasis

**DOI:** 10.1002/path.5572

**Published:** 2021-01-19

**Authors:** Keqiang Chen, Teizo Yoshimura, Xiaohong Yao, Wanghua Gong, Jiaqiang Huang, Amiran K Dzutsev, John McCulloch, Colm O'hUigin, Xiu‐wu Bian, Giorgio Trinchieri, Ji Ming Wang

**Affiliations:** ^1^ Cancer and Inflammation Program, Center for Cancer Research National Cancer Institute at Frederick Frederick MD USA; ^2^ Laboratory of Cancer and Immunometabolism, Center for Cancer Research National Cancer Institute at Frederick Frederick MD USA; ^3^ Department of Pathology and Experimental Medicine Graduate School of Medicine, Dentistry and Pharmaceutical Sciences, Okayama University Okayama Japan; ^4^ Institute of Pathology and Southwest Cancer Center Third Military Medical University Chongqing PR China; ^5^ Basic Research Program Leidos Biomedical Research, Inc Frederick MD USA; ^6^ College of Life Sciences Beijing Jiaotong University Beijing PR China

**Keywords:** Cnlp, Camp, CRAMP, epithelial cell‐derived CRAMP, myeloid cell‐derived CRAMP, colon, mucosa, proliferation, bacteria, inflammation, necrosis

## Abstract

The cathelin‐related antimicrobial peptide CRAMP protects the mouse colon from inflammation, inflammation‐associated carcinogenesis, and disrupted microbiome balance, as shown in systemic *Cnlp* ^*−/−*^ mice (also known as *Camp* ^*−/−*^ mice). However, the mechanistic basis for the role and the cellular source of CRAMP in colon pathophysiology are ill defined. This study, using either epithelial or myeloid conditional *Cnlp* ^*−/−*^ *mice*, demonstrated that epithelial cell‐derived CRAMP played a major role in supporting normal development of colon crypts, mucus production, and repair of injured mucosa. On the other hand, myeloid cell‐derived CRAMP potently supported colon epithelial resistance to bacterial invasion during acute inflammation with exacerbated mucosal damage and higher rate of mouse mortality. Therefore, a well concerted cooperation of epithelial‐ and myeloid‐derived CRAMP is essential for colon mucosal homeostasis. © 2020 The Authors. *The Journal of Pathology* published by John Wiley & Sons, Ltd. on behalf of The Pathological Society of Great Britain and Ireland.

## Introduction

The intestinal epithelium plays an essential role in host resistance against microbe‐triggered gastrointestinal diseases. Defects in barrier integrity, a principal pathological feature of mucosal inflammation, result in increased mucosal permeability, which in turn permits an unrestricted entry of harmful bacteria. Therefore, maintenance of epithelial barrier integrity is critical for protecting intestinal homeostasis [1, 2].

Under normal conditions, the colons of both human and mouse are inhabited by large numbers of bacteria [3], segregated from the mucosa by layers of mucus. In mouse colon, there are two layers of mucus containing similar protein composition with the larger gel‐forming mucin Muc2, a major component. The densely packed inner mucus layer is firmly attached to the epithelium and devoid of bacteria. In contrast, the movable outer layer is more abundant in volume, due to proteolytic cleavage of the Muc2 mucin, and is colonized by bacteria. In *Muc2* ^*−/−*^ mice, bacteria in the colon are in direct contact with epithelial cells far down in the crypts, resulting in the development of inflammation and cancer [3]. Therefore, Muc2 mucins contribute to the formation of a barrier that protects colon epithelia from direct contact by the bacteria. In addition to the mucus layer, macrophages residing in the subepithelial lamina propria of the colon mucosa also contribute to barrier integrity as sentinels for potentially harmful agents [4]. Macrophages not only possess the ability to remove dying cells in the colon by efferocytosis, but also patrol the epithelial barrier to prevent the entry and colonization of pathogens in the mucosa [5].

The colon also contains antimicrobial peptides, such as cathelin‐related antimicrobial peptide (CRAMP) in mice [6, 7] and cathelicidin antimicrobial peptide (CAMP) LL‐37 in humans [8], which are produced by epithelial [9, 10] and myeloid cells [6, 11, 12, 13, 14]. CRAMP has been shown to prevent inflammation in the colon by enhancing mucus production and reducing pro‐inflammatory cytokines. As a chemotactic agonist for a G‐protein coupled formyl peptide receptor 2 (Fpr2) in mice [15], CRAMP also promotes the healing of epithelial wounds by stimulating re‐epithelialization and angiogenesis [16, 17]. In fact, the colon of *systemic Cnlp* ^*−/−*^ mice (also known as *Camp* ^*−/−*^ mice) exhibits shortened crypts, a decreased number of goblet cells, a thinner layer of mucus, and dysbiosis [17, 18, 19]. *Systemic Cnlp* ^*−/−*^ mice are highly sensitive to chemically induced ulcerative colitis (UC) in which colon mucosa was severely damaged and infiltrated by many inflammatory cells [17], in association with markedly increased incidence of adenocarcinoma. However, despite the strong evidence of the involvement of CRAMP in protecting the integrity of the colon mucosa, the cellular sources of CRAMP in the colon and the mechanistic basis for its action remain to be elucidated.

Therefore, in this study, we investigated the contribution of CRAMP produced by colon epithelial cells and myeloid cells (mainly macrophages) to colon homeostasis and inflammatory responses by using mice with conditional *Cnlp* gene deficiency. Here, we report that while epithelial cell‐derived CRAMP plays an important role in colon epithelial cell proliferation, the production of mucus in the colon, and mucosa recovery after injury, myeloid cell‐derived CRAMP prevents inflammatory responses by harnessing bacterial over‐expansion and invasion of epithelial cells. Thus, CRAMP produced by epithelial and myeloid cells co‐operatively protects the homeostasis of the colon.

## Materials and methods

### Mice

Myeloid cell‐specific *Cnlp* ^*−/−*^ mice (*LysMCre* ^*+*^ *Cnlp* ^*F/F*^) and epithelial cell‐specific *Cnlp* ^*−/−*^ mice (*VillinMCre* ^*+*^ *Cnlp* ^*F/F*^) were generated as previously described [17, 20]. In brief, homozygous *Cnlp* floxed mice (*Cnlp* ^*F/F*^) were crossed to either LysMCre or VillinCre mice on a C57BL/6 genetic background to generate myeloid cell‐ or intestinal epithelial cell‐specific *Cnlp* ^*−/−*^ mice. Systemic *Fpr2* ^−/−^ and *Cnlp* ^−/−^ mice were generated as described previously [21, 22]. Myeloid cell‐specific *Fpr2*‐deletion (*LysMCre* ^*+*^ *Fpr2* ^−/−^) and epithelial cell‐specific *Fpr2‐*deletion (*VillinMCre* ^*+*^ *Fpr2* ^−/−^) mice were generated as described [20]. *Fpr2/Cnlp double* (*dl*)^−/−^ mice were generated by crossing *Fpr2* ^*−/−*^ and *Cnlp* ^*−/−*^ mice. Mice used in the experiments were 8–12 weeks old and were allowed free access to standard laboratory chow/tap water. All animals were housed in an air‐conditioned room with controlled temperature (22 ± 1 °C), humidity (65–70%), and day/night cycle (12 h light, 12 h dark). All animal procedures were governed by the US NIH Guide for the Care and Use of Laboratory Animals [23] and were approved by the Animal Care and Use Committee of the NCI‐Frederick, National Institutes of Health.

### Induction of colitis

Colitis was induced by administration of 3% dextran sulfate sodium (DSS; 4,000 kDa) in drinking water for 5 days. The colons were then harvested with or without a subsequent 7 days of normal water intake. Alternatively, the mice were given 3% DSS in drinking water for 3 days, followed by 4 days of normal water intake.

### Histology and immunohistochemistry

Paraffin sections (5 μm) of mouse colon were attached to poly‐l‐lysine‐coated glass slides (Thermo Scientific, Dreieich, Germany). After incubation at 60 °C for 1 h, the slides were dewaxed and hydrated using stepwise 100% xylene, 100% ethanol, followed by distilled water containing decreasing concentrations of ethanol. Colon sections were stained with hematoxylin and eosin (H&E) and periodic acid–Schiff (PAS) (Cat #: 395B‐1KT; Sigma‐Aldrich, St Louis, MO, USA). Between five and eight morphologically well‐oriented crypt regions were randomly chosen per colon section and their length was measured using ImageJ software (NIH, Bethesda, MD, USA). For CRAMP staining, H_2_O_2_ was used to suppress endogenous peroxidase activity and sections were blocked in 10% normal rat serum with 1% BSA in TBS for 2 h at room temperature before incubation with a primary antibody to mouse CRAMP diluted 1:100 in TBS with 1% BSA. An HRP‐conjugated secondary antibody in 0.3% H_2_O_2_ in TBS was used and binding was detected using DAB solution (Cat #: 34002; Thermo, Rockford, IL, USA). Sections were counterstained with hematoxylin. The severity of colitis was determined by histopathological change index (HCI). The scoring system includes scores 0–5 based on (1) the extent of colon tissue affected, (2) the extent of crypt damage, and (3) the quantity and dimension of inflammatory cell infiltration [24], as detailed in supplementary material, Table S1.

### Detection of CRAMP produced by mouse macrophages

The mouse macrophage‐derived cell line RAW 264.7 (ATCC® TIB‐71; ATCC, Manassas, VA, USA) was seeded in 35 mm dishes with 14 mm coverslips on the bottom (Cat #: P35G‐1.0‐14‐C; MatTek, Ashland, MA, USA) at 1 × 10^6^ cells per dish. The macrophages were cultured without or with inactivated *E. coli* (*E. coli* K‐12, Cat #: 155068; Carolina Biological Supply Company, Burlington, NC, USA) at 1 × 10^7^ CFU per well (MOI = 100). Twenty hours later, cells were washed with PBS and fixed with 4% neutral buffered formalin for 10 min. Cells were stained with anti‐mouse CRAMP antibody (Ab) (Cat #: sc‐166055, 1/100 dilution; Santa Cruz Biotechnology, Inc, Dallas, TX, USA) followed by a biotinylated anti‐Ig secondary Ab and streptavidin‐PE. DAPI was used to stain nuclei. For inactivation of *E. coli*, the concentration of live *E. coli* was diluted to 2 × 10^6^ CFU/ml and 0.4% formalin (by volume ratio) was added, followed by incubation with continuous shaking (200 rpm) in a shaker incubator at 37 °C overnight.

### Immunofluorescence

Immunofluorescence analyses were performed on fresh‐frozen, OCT‐embedded and sectioned colon tissue specimens (10 μm thick, colons from 6–8 mice per group). For detection of Ki67 and Muc2 expression, primary Ki67 Ab (Cat #: ab16667, 1/200 dilution; Abcam, Cambridge, MA, USA) and Muc2 Ab (Cat #: ab272692, 1/1000 dilution; Abcam) were used. For detection of CRAMP expression in colon epithelial cells or in colon macrophages, primary anti‐CRAMP (Cat #: sc‐166 055, 1/100 dilution; Santa Cruz Biotechnology, Inc) and anti‐EpCAM (Cat #: ab71916, 1/100 dilution; Abcam) or anti‐MOMA‐2 (Cat #: sc‐59 332, 1/100 dilution; Santa Cruz Biotechnology, Inc) were used. For Fpr2 expression, primary anti‐mouse Fpr2 Ab (Cat #: sc‐57141, 1/100 dilution; Santa Cruz Biotechnology, Inc) was used. For detection of macrophages in colon mucosa, primary antibodies against MOMA‐2 (Cat #: sc‐59332, 1/100 dilution; Santa Cruz Biotechnology, Inc) and F4/80 (Cat #: ab6640, 1/100 dilution; Abcam) were used. Frozen sections of colon tissue were fixed in 4% neutral buffered formalin for 5 min and stained with primary antibodies or an isotype control Ab followed by a biotinylated anti‐Ig secondary antibody and streptavidin‐PE or ‐FITC. DAPI was used to stain nuclei. Multiple (4–8) viewing fields on each slide were randomly selected and images acquired using an Olympus DP camera with CellSens (Ver. 1.17) imaging software. The results were quantitated by NIH ImageJ software.

### Heat map analysis of fecal bacterial population

Fecal pellets were harvested from mice at 12 weeks of age. Fecal DNA was prepared using a DNA Stool Mini Kit (Cat #: 51604; QIAGEN, Germantown, MD, USA) and sequenced. In brief, the V4 fragment of 16S rDNA was amplified by PCR using primers 515F: 5'‐GTGCCAGCMGCCGCGGTAA‐3' and 806R: 5'‐GGACTACHVGGGTWTCTAAT‐3' flanked by p5 and p7 Illumina Sequencing adaptors (p5 and p7), barcode (i5 and i7), pad (to optimize melting temperature), and a link sequence. PCR products were purified and normalized using a SequalPrep Normalization Plate Kit (Invitrogen). Sequencing was performed using Mothur v.1.30.0. as described in the MiSeq 16S standard operating procedure protocol. All sequencing was performed using the National Institutes of Health Biowulf Cluster. Statistical analysis was performed using ANOVA and *P* values were corrected for multiple comparisons using the *q*‐value test (0.1) [17].

### Wound healing assay

CT26 mouse colon carcinoma epithelial cells (ATCC) were plated in six‐well plates at a density of 1.5 × 10^5^ cells per well. When confluent, a scratch was made using a sterile 200‐μl pipette tip. Synthetic CRAMP was purchased from Hycult Biotech, Wayne, PA, USA (Cat #: HC1106, with amino acid sequence ISRLAGLLRKGGEKIGEKLKKIGQKIKNFFQKLVPQPE) and stored at −20 °C in aliquots at 30 mg/ml in PBS before use. CRAMP was then diluted in PBS to the desired concentrations before use. Cells were treated with medium (10% FBS DMEM) or 10% FBS DMEM with CRAMP. Some parallel wells were pretreated with a selective Fpr2 antagonist, WRW4 (Cat #: 2262; TOCRIS, Minneapolis, MN, USA), for 1 h followed by CRAMP. Images of scratch wounds were taken with an inverted microscope (BX71; Olympus Corporation, Japan). The distances that the cells migrated from the edges towards the center of the wounds were determined after 20 h of incubation.

### Detection of bacteria attaching to and invading colonic mucosa

Fresh‐frozen, OCT‐embedded tissue from 6–8 mice per group was cryosectioned (10 μm). Sections were fixed in 4% neutral buffered formalin for 5 min and bacteria were detected using a Bacterial Gram Staining Kit (Cat #: ab253409; Abcam) following the manufacturer's protocol. Sections were dehydrated in absolute alcohol, cleared in xylene, and then mounted in a synthetic resin.

### Immunoblotting

CT26 mouse colon carcinoma epithelial cells were grown in 60‐mm dishes to sub‐confluency and then cultured for 3 h in FCS‐free medium. After treatment with CRAMP (Cat #: HC1106; Hycult Biotech, Wayne, PA, USA), the cells were lysed with 1× SDS sample buffer [62.5 mm Tris–HCl (pH 6.8), 2% SDS, 10% glycerol, and 50 mm dithiothreitol], then sonicated for 15 s and heated at 100 °C for 5 min. The cell lysate was centrifuged at 11 269 × *g* at 4 °C for 5 min, and protein concentrations of the supernatants were measured using a DC Protein Assay (Bio‐Rad Laboratories Inc, Hercules, CA, USA). The lysates with titrated proteins were electrophoresed through 10% SDS‐PAGE precast gels (Invitrogen) under reducing conditions and then transferred onto ImmunoBlot polyvinylidene membranes (Bio‐Rad Laboratories Inc), which were blocked with 5% nonfat dried milk. Phospho‐specific Abs were used to detect phosphorylated p38 (Cat #: 9211, 1/1000 dilution; Cell Signaling Technology, Beverly, MA, USA) and ERK1/2 (Cat #: 9101, 1/1000 dilution; Cell Signaling Technology) according to the manufacturer's instructions. After incubation of the membranes with an anti‐rabbit IgG horseradish peroxidase‐conjugated secondary Ab (Cat #: 7074, 1/3000 dilution; Cell Signaling Technology), protein bands were detected with a Super Signal Chemiluminescent Substrate (Cat #: 34076; Thermo Scientific, Rockford, IL, USA) and the band intensities were quantitated using a G‐BOX GeneSnap system (SYNGENE, Frederick, MD, USA). The membranes were stripped with Restore Western Blot Stripping Buffer (Cat #: 21059; Thermo Fisher, Waltham, MA, USA) followed by incubation with specific Abs. The membranes were stripped with Restore Western Blot Stripping Buffer (Cat#: 21059, ThermoFisher, Waltham, MA, USA) followed by incubation with specific antibodies against total p38 (Cat #: 9212, 1/1000 dilution, Cell Signaling Technology), ERK1/2 (Cat#: 9102, 1/1000 dilution, Cell Signaling Technology), IκB‐α (Cat#: 9247, 1/1000 dilution, Cell Signaling Technology) for detection of p38, ERK1/2 and IκB‐α.

### Enzyme‐linked immunosorbent assay (ELISA)

Mouse peripheral blood was collected into heparin‐coated tubes. After centrifugation, plasma was collected and stored at −80 °C for later analysis. The levels of IL1‐β, IL‐6, and IL‐10 were measured by ELISA (IL‐1β ELISA Kit: Cat # BMS6002; IL‐6 ELISA Kit: Cat # BMS603‐2; IL‐10 ELISA Kit, Cat # 88‐7105‐22; Thermo Fisher Scientific, Waltham, MA USA).

### Statistics

All experiments were performed at least three times with three replicate samples. Statistical analysis was performed using GraphPad Prism (GraphPad Software, San Diego, CA, USA) and Student's *t‐*test (for two groups) or one‐way ANOVA with Kruskal–Wallis tests (for more than two groups). Log‐rank Mantel–Cox tests were used for comparison of survival curves. Data with error bars show mean ± SEM and *P* values less than 0.05 were considered statistically significant.

## Results

### The expression of CRAMP by colon epithelial cells and resident macrophages

It is well known that the entire intestinal epithelium is replaced every 2–3 days in mice (versus 3–5 days in humans); therefore, crypt cells go through a process of generation, differentiation, migration, and turnover. The epithelial cells in crypts may be divided into stem cells, proliferating (differentiating) cells, and mature (differentiated) cells [25, 26]. Our double staining of the colon detected CRAMP in Ki67^+^ epithelial cells located in the lower and middle regions of the crypts (supplementary material, Figure S1A,B), which was released to the surface of the colon mucosa to mix in a layer of Muc2 secreted by goblet cells, overlaying the epithelium (supplementary material, Figure S2A). These results indicate that epithelial precursor cells (differentiating cells) in the crypts of naïve mice express CRAMP. In acute inflammation, CRAMP secretion into the intestinal lumen was increased, resulting in a much reduced CRAMP content remaining in epithelial cells (supplementary material, Figure S2B). In chronic inflammation, the production of CRAMP was also increased, and CRAMP‐containing cells appeared in the differentiated region of colon crypts (supplementary material, Figure S2C). We also found that the CRAMP production was increased in mouse macrophages (RAW264.7) 20 h after stimulation of inactivated *E. coli* (supplementary material, Figure S2D). These results indicate that epithelial cells and macrophages express CRAMP.

We further examined the expression of CRAMP by epithelial cells and myeloid cells (mainly macrophages) in the mouse colon mucosa. In the colon epithelial cells of *epithelial Cnlp* ^*−/−*^ mice, CRAMP was not detectable (supplementary material, Figure S3A); in contrast, CRAMP was readily detected in macrophages which were scattering the colon mucosa (supplementary material, Figure S3B). Conversely, in the colon of *myeloid Cnlp* ^*−/−*^ mice, the macrophages scattering the colon mucosa were negative for CRAMP, whereas epithelial cells were positive (supplementary material, Figure S3C,D). Thus, CRAMP as an endogenous antimicrobial peptide is produced by both colon epithelial cells and macrophages distributed in the mucosa.

### Contribution of epithelial cell‐derived CRAMP to Muc2 production in the colon

**Figure 1:**
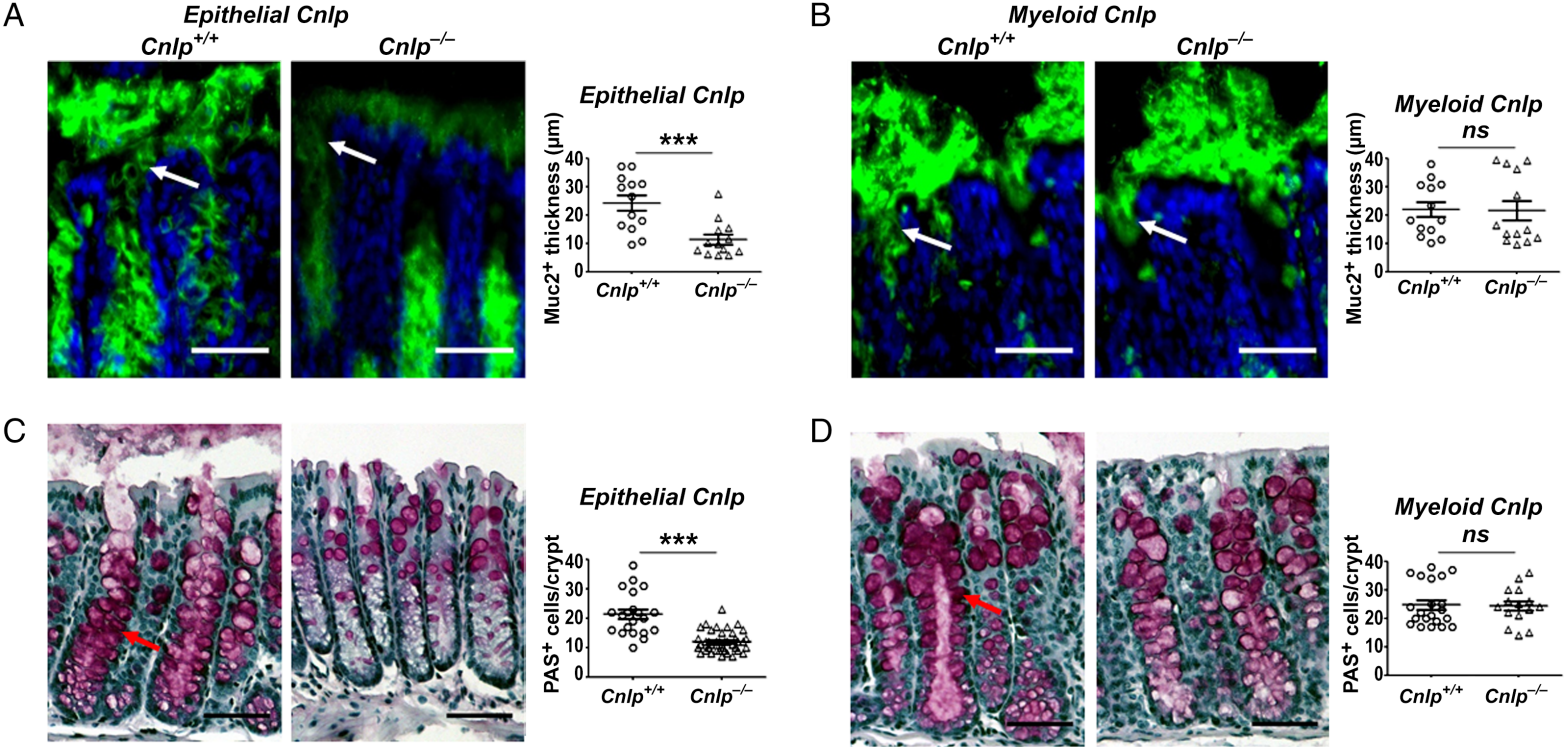
Contribution of epithelial cell‐derived CRAMP to Muc2 production in the colon. (A) Reduced Muc2 expression in the colon mucosa of *epithelial Cnlp* ^*−/−*^ mice. Left panel: representative images are shown. Green: Muc2; blue: DAPI. White arrow: Muc2^+^ materials released onto the surface of colonic mucosa. Scale bar = 50 μm. Right panel: measurement of Muc2 thickness on the surface of colon mucosa. *n* = 13 per group; ***p < 0.001. (B) Muc2 expression in the colonic mucosa of *myeloid Cnlp* ^*−/−*^ mice. Left panel: representative images are shown. Green: Muc2; blue: DAPI. White arrow: Muc2^+^ materials released onto the surface of the mucosa. Scale bar = 50 μm. Right panel: measurement of Muc2^+^ thickness on the surface of colonic mucosa, *n* = 20 per group. (C) Reduced PAS^+^ cells in the colon crypts of *epithelial Cnlp* ^*−/−*^ mice. Left panels: representative images are shown. Red arrow: PAS^+^ cells. Scale bar = 100 μm. Right panel: PAS^+^ cell number per colonic crypt. *n* = 21–30 per group; ***p < 0.001. (D) No change of PAS^+^ cell number in the colonic crypts of *myeloid Cnlp* ^*−/−*^ mice. Left panels: representative images are shown. Red arrow: PAS^+^ cells. Scale bar = 100 μm. Right panel: PAS^+^ cells per colon crypt. *n* = 15–21 per group.

Since human LL‐37 stimulates mucin production in the gut [27] and *Cnlp* ^*−/−*^mice contained a thinner layer of mucus in the colon [18], we investigated whether epithelial cell‐ or macrophage‐derived CRAMP promoted Muc2 production. As shown in Figure 1A, the thickness of Muc2 on the colon mucosa of *epithelial Cnlp* ^*−/−*^ mice was significantly reduced compared with the *epithelial Cnlp* ^*+/+*^ mice. In contrast, the thickness of Muc2 on the colon of *myeloid Cnlp* ^*−/−*^ mice did not show any reduction compared with *myeloid Cnlp* ^*+/+*^ mice (Figure 1B). Thus, epithelial cell‐derived CRAMP stimulates Muc2 production in the colon.

Further study revealed that *epithelial Cnlp* ^*−/−*^ mice had fewer PAS^+^ goblet cells (which synthesize and secrete bioactive components of mucus) compared with *epithelial Cnlp* ^*+/+*^ mice (Figure 1C). In contrast, the number of PAS^+^ goblet cells in the colon crypt of *myeloid Cnlp* ^*−/−*^ mice did not show any reduction compared with *myeloid Cnlp* ^*+/+*^ mice (Figure 1D), indicating that the reduction of PAS^+^ goblet cells in the colon crypts of *epithelial Cnlp* ^*−/−*^ mice is the cause of reduced Muc2 production.

**Figure 2:**
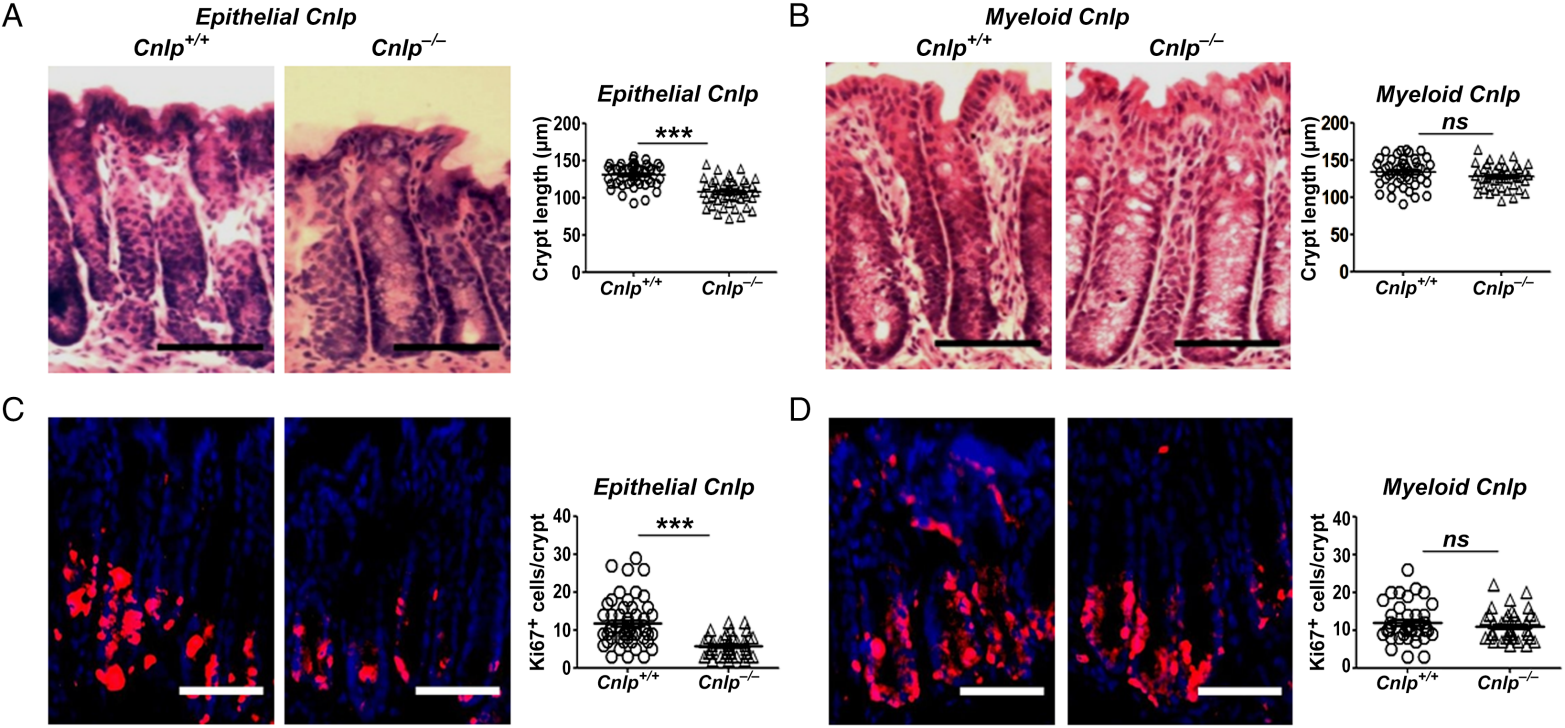
Reduced epithelial cell proliferation in the colon of *epithelial Cnlp* ^*−/−*^mice. (A) Shortened colon crypts in *epithelial Cnlp* ^*−/−*^ mice. Left panel: representative images to show H&E staining of colon sections from naïve *epithelial Cnlp* ^*−/−*^ and *epithelial Cnlp* ^*+/+*^ mice. Scale bar = 50 μm. Right panel: colonic crypt length (*n* = 8 per group). ***p < 0.001. (B) No change in the length of colonic crypts of *myeloid Cnlp* ^*−/−*^mice compared with *myeloid Cnlp* ^*+/+*^mice. Left panel: representative images are shown. Scale bar = 50 μm. Right panel: quantitative analysis of colon crypt length of naïve *myeloid Cnlp* ^*−/−*^ and *myeloid Cnlp* ^*+/+*^mice. *n* = 8 per group. (C) Reduced numbers of Ki67^+^ cells in the colonic crypts of *epithelial Cnlp* ^*−/−*^ mice. Left panels: representative images of immunofluorescence for Ki67 are shown. Red: Ki67^+^ cells; blue: DAPI. Scale bar = 100 μm. Right panel: numbers of Ki67^+^ cells per crypt (*n* = 8 per group). ***p < 0.001. (D) No change in the numbers of Ki67^+^ cells in the colon crypts of *myeloid Cnlp* ^*−/−*^ mice compared with *myeloid Cnlp* ^*+/+*^ mice. Left panels: red: Ki67^+^ cells; blue: DAPI. Scale bar = 60 μm. Right panel: quantitative analysis of Ki67^+^ cells per crypt (*n* = 8 per group). ns = no significance between *myeloid Cnlp* ^*−/−*^ mice and *myeloid Cnlp* ^*+/+*^ mice.

We previously reported the shortening of the crypt length in the colon of *systemic Cnlp* ^*−/−*^ mice [17]. This phenotype was also observed in the colon of *epithelial Cnlp* ^*−/−*^ mice (Figure 2A), but not *myeloid Cnlp* ^*−/−*^ mice (Figure 2B). The crypts in the colon of *epithelial Cnlp* ^*−/−*^ mice contained fewer Ki67^+^ cells, suggesting compromised epithelial cell proliferation (Figure 2C,D). These results indicated that CRAMP derived from colonic epithelial cells is responsible for the normal crypt length, goblet cell number, and Muc2 production.

### Increased bacterial attachment to colon epithelia in *myeloid Cnlp* ^*−/−*^ mice

**Figure 3:**
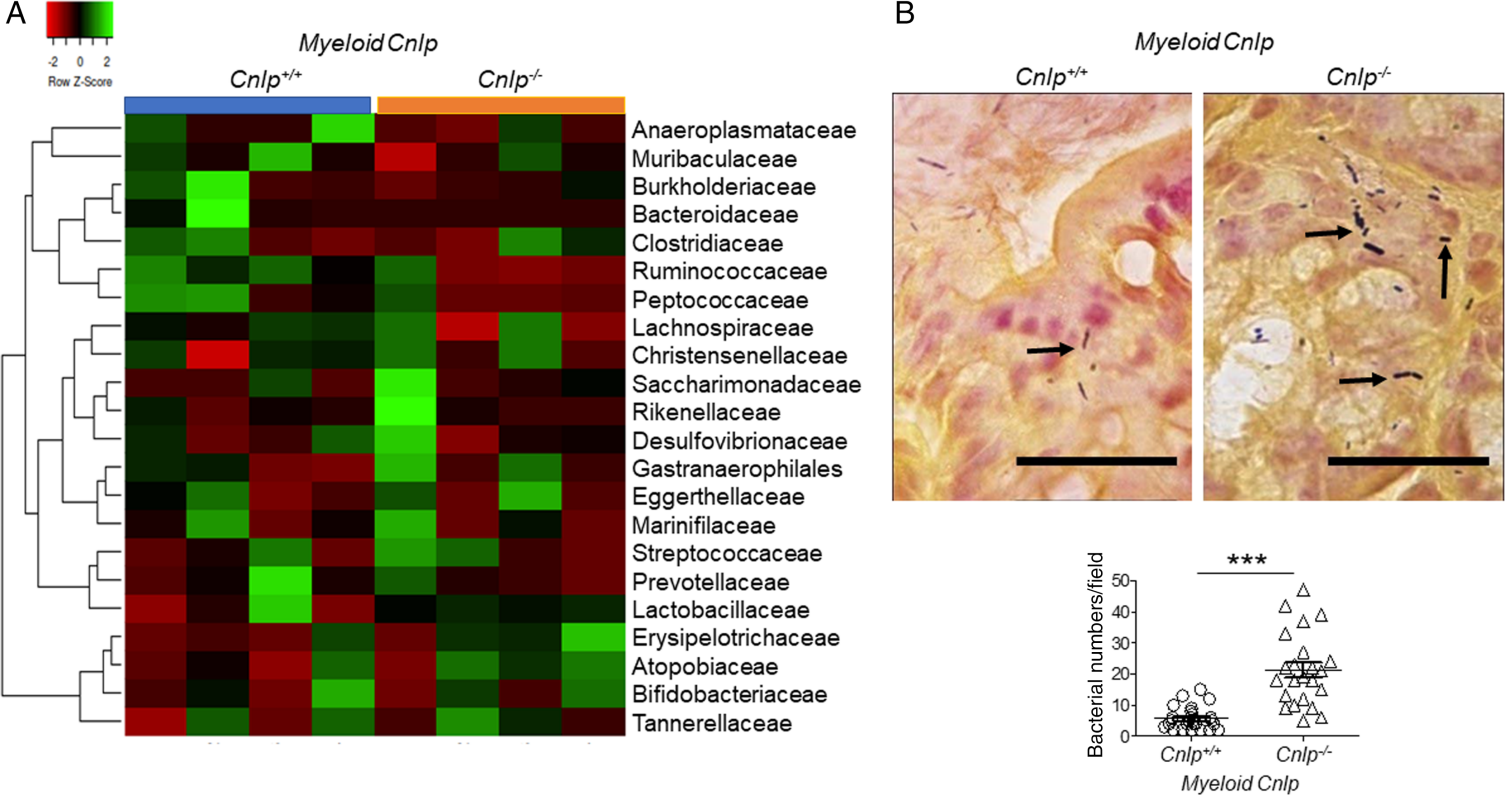
Increased bacterial attachment to colonic epithelium of naïve *myeloid Cnlp* ^*−/−*^ mice. (A) Myeloid cell‐derived CRAMP deficiency does not alter the microbiota composition in the colon of *myeloid Cnlp* ^*−/−*^ mice co‐housed with *myeloid Cnlp* ^*+/+*^ mice. Fecal bacterial DNA was isolated from four *myeloid Cnlp* ^*−/−*^ mice and four *myeloid Cnlp* ^*+/+*^ mice at the age of 12 weeks. 16S sequence frequencies were analyzed by 16S amplicon high‐throughput sequencing of fecal microbiota. Data are shown as a heat map of classified sequences. (B) Increased number of bacteria attached to the colon mucosa of *myeloid Cnlp* ^*−/−*^ mice. Upper panels: representative images are shown. Black arrow: bacteria. Scale bar = 30 μm. Lower panel: counts of attached bacteria in the colon mucosa of *myeloid Cnlp* ^*−/−*^ and *myeloid Cnlp* ^*+/+*^ mice. *n* = 23–28 fields per group; eight mice per group. ***p < 0.001.

We then examined the role of CRAMP in maintaining the balance of the colon microbiome. A heat map of the microbiome composition (Figure 3A) revealed no statistical significance in at least 22 bacterial strains in the feces comparing *myeloid Cnlp* ^*−/−*^ mice and *myeloid Cnlp* ^*+/+*^ littermates at the age of 12 weeks, although there was a tendency for an increase in eight bacteria strains including *Bacteroides*, *Alistipes*, *Anaerostips*, *Gastranaerophilales*, *Dubosiella*, *Turicibacter*, *Saccharimonadaceae*, and *Akkermansia*, and a reducing abundance of five bacterial strains including *Lactobacillus*, *Lachnospiraceae*, *Oscillibacter*, *Parasutterella*, and *Anaeroplasma*. However, the number of bacteria adhering to the epithelium of naïve *myeloid Cnlp* ^*−/−*^ mice was significantly increased compared with *myeloid Cnlp* ^*+/+*^ littermates (Figure 3B).

Similarly, a heat map of the microbiome composition (supplementary material, Figure S4A) revealed no statistical significance in at least 21 bacterial strains in the feces from naïve *epithelial Cnlp* ^*−/−*^ mice versus *epithelial Cnlp* ^*+/+*^ littermates, although there was an increasing tendency in six bacterial strains including *Bacteroidaceae*, *Gastranaerophilales*, *Clostridiales*, *Lachnospiraceae*, *Ruminococcaceae*, and *Desulfovibrionaceae*, and a reducing abundance in four bacterial strains including *Muribaculaceae*, *Prevotellaceae*, *Lactobacillaceae*, and *Peptostreptococcaceae*. Interestingly, the number of bacteria adhering to the epithelium of naïve *epithelial Cnlp* ^*−/−*^ mice was not changed compared with *epithelial Cnlp* ^*+/+*^ littermates (supplementary material, Figure S4B). These results indicate that the co‐housing of *Cnlp* ^*−/−*^ mice and *Cnlp* ^*+/+*^ littermates results in the balance of the microbiome composition in the colon [17] and furthermore reveals that myeloid cell (mainly macrophages)‐derived CRAMP plays an important role in preventing bacterial attachment to colonic epithelia to maintain normal mucosal barrier function.

Interestingly, *epithelial Cnlp* ^*−/−*^ mice (compared with *myeloid Cnlp* ^*−/−*^ mice) had reduced levels of five bacterial strains including *Muribaculaceae* (*myeloid Cnlp* ^*−/−*^ 4987.25 versus *epithelial Cnlp* ^*−/−*^ 394.5, *p* < 0.001), *Blautia* (*myeloid Cnlp* ^*−/−*^ 32.75 versus *epithelial Cnlp* ^*−/−*^ 13.5, *p* < 0.05), *Roseburia* (*myeloid Cnlp* ^*−/−*^ 13.25 versus *epithelial Cnlp* ^*−/−*^ 0.25, *p* < 0.05), *Peptococcaceae* (*myeloid Cnlp* ^*−/−*^ 85.25 versus *epithelial Cnlp* ^*−/−*^ 32, *p* < 0.05), and *Parasutterella* (*myeloid Cnlp* ^*−/−*^ 345 versus *epithelial Cnlp* ^*−/−*^ 106.75, *p* < 0.05), indicating that *Cnlp* deficiency in different cell types in the colon has a differential effect on the fecal bacterial composition.

### Higher sensitivity of *myeloid Cnlp* ^*−/−*^ mice to chemically induced colitis

Since *systemic Cnlp* ^*−/−*^ mice are highly sensitive to chemically induced colitis [17], we compared the ability of epithelial‐derived and myeloid‐derived CRAMP to protect the colon from an inflammatory challenge.

**Figure 4:**
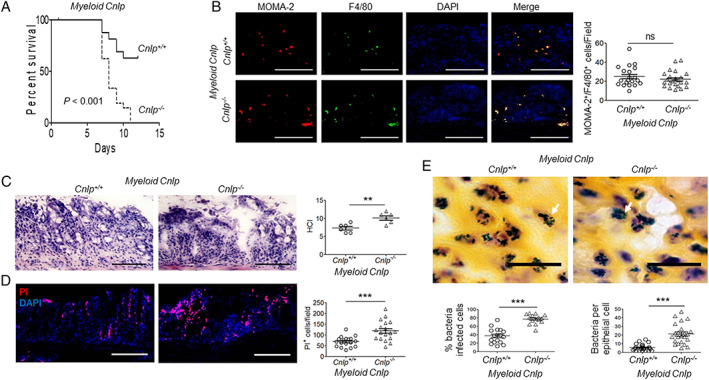
Higher sensitivity of *myeloid Cnlp* ^*−/−*^ mice to chemically induced colitis. (A) Reduced survival of *myeloid Cnlp* ^*−/−*^ mice after administration of 3% DSS in drinking water for 5 days followed by an additional 7 days of normal water intake. ***p < 0.001. (B) No significant changes of macrophage numbers in the colonic mucosa of *myeloid Cnlp* ^*−/−*^ mice compared with *myeloid Cnlp* ^*+/+*^ mice after DSS treatment for 5 days. Left panels: representative images of MOMA‐2^+^/F4/80^+^ macrophages in colonic mucosa. *n* = 20 fields per group. Right panel: counts of MOMA‐2^+^/F4/80^+^ macrophages per field. ns = no significant difference. (C) Increased damage for colon mucosa in *myeloid Cnlp* ^*−/−*^ mice treated with DSS for 5 days. Left panels: representative images showing colon sections stained with H&E. Scale bar = 100 μm. Right panel: the histopathological change index (HCI). *n* = 6 per group. **p < 0.01. (D) Increased colon epithelial cell death in *myeloid Cnlp* ^*−/−*^ mice treated with DSS for 5 days. Left panels: representative images showing PI^+^ cells in colon mucosa. Scale bar = 100 μm. Right panel: counts of PI^+^ cells per field. *n* = 17 per group. ***p < 0.001. (E) Increased numbers of bacteria invaded the colon epithelial cells of *myeloid Cnlp* ^*−/−*^ mice. Upper panels: representative images showing bacteria that have entered colon epithelial cells. White arrow: bacteria in epithelial cells. Scale bar = 30 μm. Lower panel: left: quantitative analysis of the percentage of infected epithelial cells in *myeloid Cnlp* ^*−/−*^ mice and *myeloid Cnlp* ^*+/+*^ mice; *n* = 8 per group. ***p < 0.001. Right: counts of the numbers of bacteria per colonic epithelial cells in *myeloid Cnlp* ^*−/−*^ mice and *myeloid Cnlp* ^*+/+*^ mice. *n* = 8 per group. ***p < 0.001.

Mice were treated with DSS for 5 days to disrupt the integrity of colon mucosa, enabling the invasion of bacteria that result in acute colitis. As shown in Figure 4A, all *myeloid Cnlp* ^*−/−*^ mice died by day 11 after DSS intake with shortening of the colon length at day 5, an indication of inflammation and scarring (supplementary material, Figure S5A). The numbers of MOMA‐2^+^ and F4/80^+^ macrophages were similar in the colon tissues of *myeloid Cnlp* ^*−/−*^ mice and *myeloid Cnlp* ^*+/+*^ littermates after DSS treatment for 5 days (Figure 4B). However, the pathological changes (Figure 4C) and epithelial cell necrosis (Figure 4D) and the number of epithelial cells invaded by bacteria were significantly increased in *myeloid Cnlp* ^*−/−*^ mice compared with *myeloid Cnlp* ^*+/+*^ littermates (Figure 4E).

**Figure 5:**
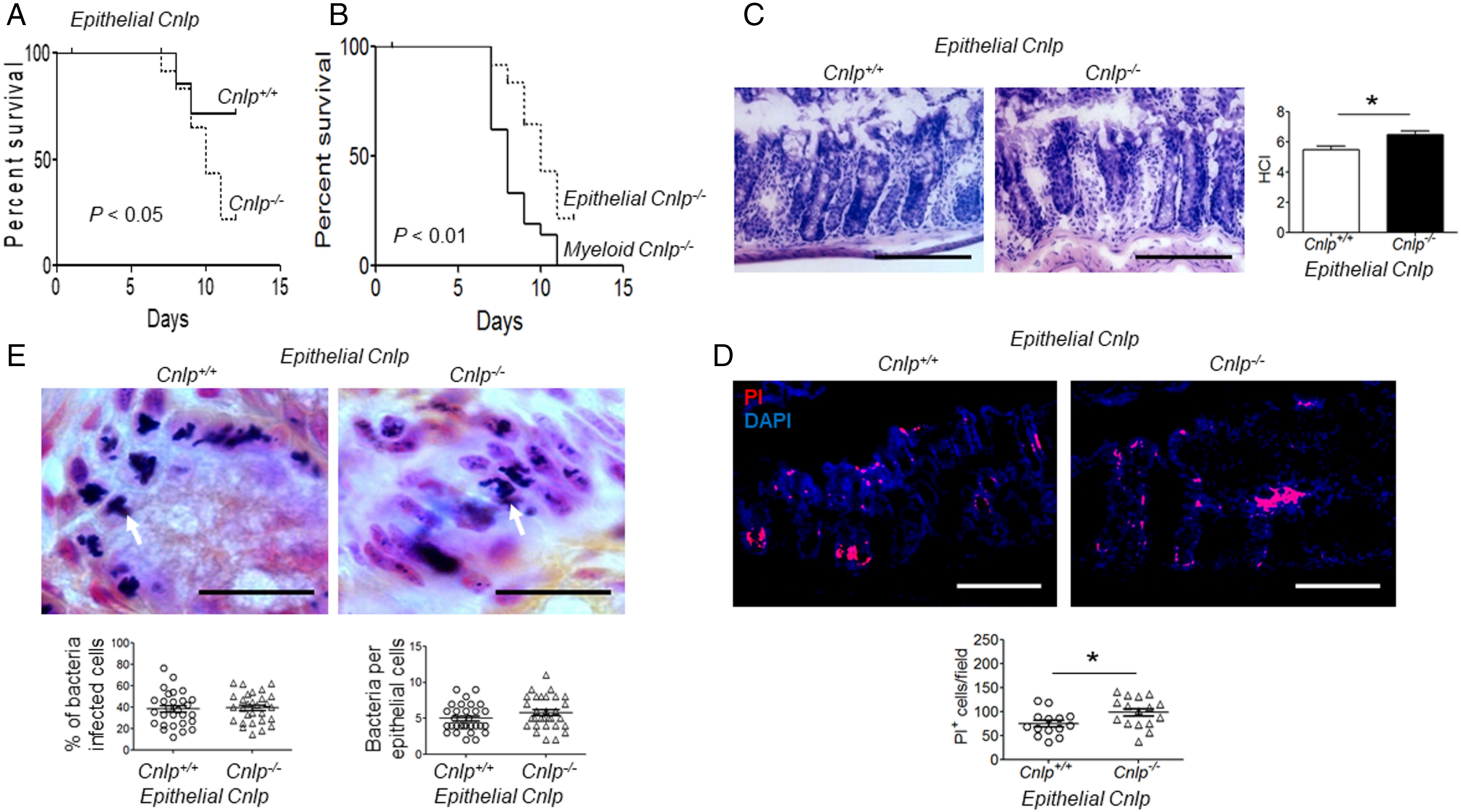
Response of *epithelial Cnlp* ^*−/−*^ mice to chemically induced colitis. Mice were given 3% DSS in drinking water for 5 days followed by an additional 7 days of normal water intake. (A) Reduced survival of *epithelial Cnlp* ^*−/−*^ mice compared with *epithelial Cnlp* ^*+/+*^ mice. *p < 0.05. (B) Reduced survival of *myeloid Cnlp* ^*−/−*^ mice compared with *epithelial Cnlp* ^*−/−*^ mice. *p < 0.01. (C) Mild damage in the colonic mucosa of *myeloid Cnlp* ^*−/−*^ mice treated with DSS for 5 days. Left panels: representative images showing colon sections stained with H&E, Scale bar = 100 μm. Right panel: histopathological change index (HCI). *n* = 6 per group. *p < 0.05. (D) Increased colonic epithelial cell death in *epithelial Cnlp* ^*−/−*^ mice treated with DSS for 5 days. Upper panels: representative images showing increased PI^+^ cell number. Scale bar = 100 μm. Lower panel: counts of PI^+^ cells per field. *n* = 14–16 per group. *p < 0.05. (E) Similar numbers of bacteria entered the colon epithelial cells of *epithelial Cnlp* ^*−/−*^ mice and *epithelial Cnlp* ^*+/+*^ mice. Upper panels: representative images are shown. White arrow: bacteria in epithelial cells. Scale bar = 30 μm. Lower panel: left: similar percentage of infected epithelial cells in *epithelial Cnlp* ^*−/−*^ mice compared with *epithelial Cnlp* ^*+/+*^ mice. Right: similar numbers of bacteria invaded the colon epithelial cells of *epithelial Cnlp* ^*−/−*^ mice and *epithelial Cnlp* ^*+/+*^ mice. *n* = 8 per group.

In contrast to the more rapid death of *myeloid Cnlp* ^*−/−*^ mice with colitis, 70% of *epithelial Cnlp* ^*−/−*^ mice died by day 12 (Figure 5A), a mortality significantly reduced compared with *myeloid Cnlp* ^*−/−*^ mice (Figure 5B) after DSS intake, with slight shortening of the colon length at day 5 (supplementary material, Figure S5B) with mild mucosal damage (Figure 5C,D). Under microscopy, although increased invasion of bacteria into epithelial cells of *epithelial Cnlp* ^*−/−*^ mice was also observed, there was no difference compared with *epithelial Cnlp* ^*+/+*^ littermates (Figure 5E). Therefore, CRAMP produced by myeloid cells (mainly macrophages) plays an important role in protecting colonic epithelial cells from bacterial invasion in acute colitis.

### Contribution of epithelial cell‐derived CRAMP to colon crypt growth

**Figure 6:**
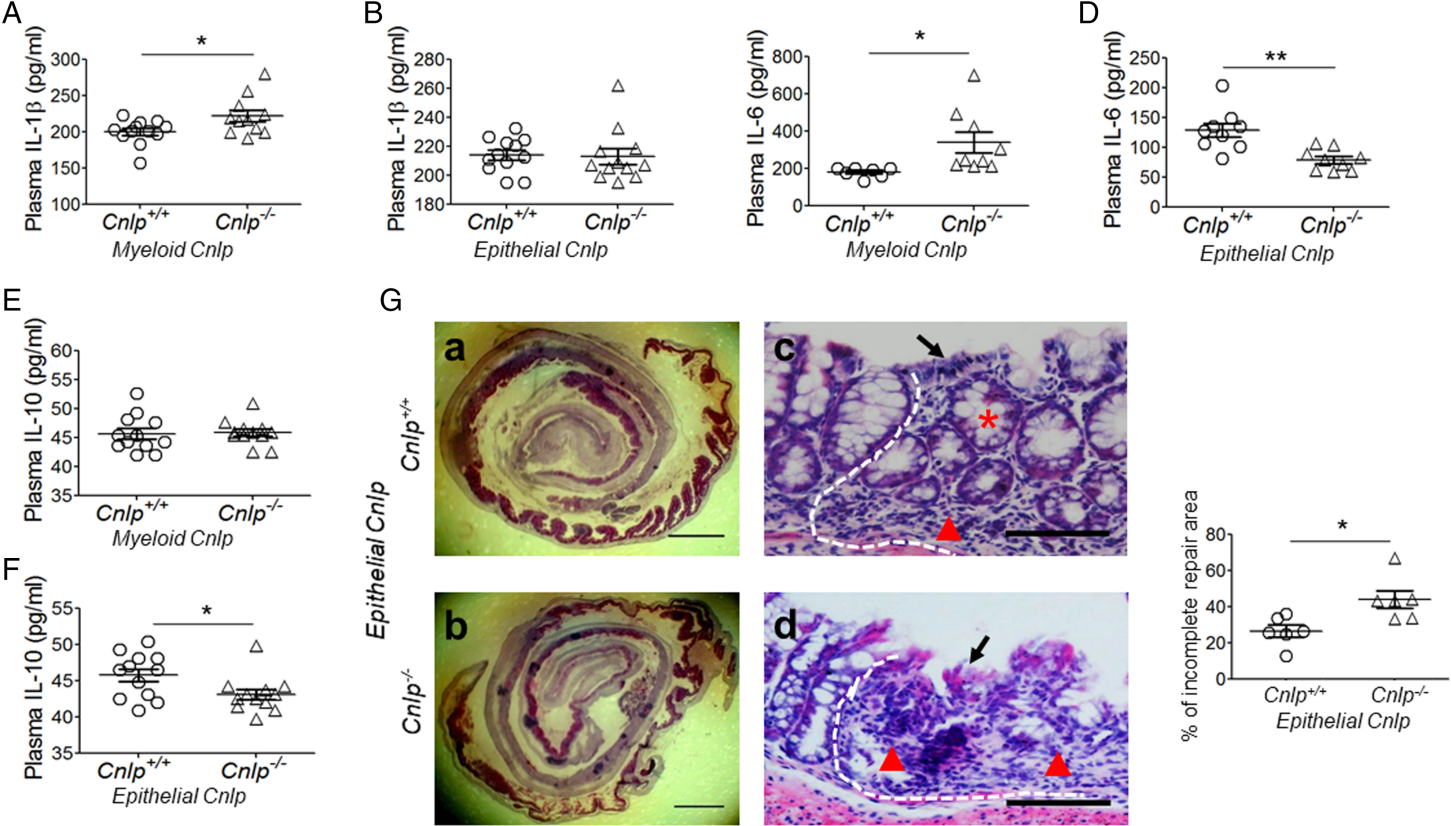
Reduced plasma levels of IL‐6 and IL‐10 and colon mucosa recovery in *epithelial Cnlp* ^*−/−*^ mice. Mice were given 3% DSS in drinking water for 5 days and then the plasma was harvested. The plasma levels of IL‐1β, IL‐6, and IL‐10 were measured by ELISA. (A, B) Plasma levels of IL‐1β; (C, D) plasma levels of IL‐6; and (E, F) plasma levels of IL‐10. *n* = 9–13 per group. *p < 0.05, **p < 0.01. (G) Reduced recovery of the colonic mucosa of *epithelial Cnlp* ^*−/−*^ mice. Mice were given 3% DSS in drinking water for 3 days followed by an additional 4 days of normal water intake. Left panels: representative images showing reduced recovery area of the colonic mucosa in *epithelial Cnlp* ^*−/−*^ mice. (a, b) Whole colon Swiss roll sections, H&E staining. Scale bar = 30 mm. (c, d) *Epithelial Cnlp* ^*−/−*^ mice showed reduced re‐epithelialization in the ulcerative focus of colon mucosa. The ulcerative focus is on the right side of the white dotted line; the normal colon mucosa is on the left. H&E staining. Scale bar = 50 μm. Black arrows: epithelial cells; red star: neoformative epithelial cells; red triangle: inflammatory lesions. Right panel: the percentage of non‐recovery area. *n* = 6 per group. *p < 0.05.

Since IL‐1β is a pro‐inflammatory cytokine which may contribute to the pathogenesis of inflammatory bowel disease (IBD) [28, 29, 30], while IL‐6 and IL‐10 are associated with colon epithelial cell proliferation [31, 32], we investigated the influence of CRAMP deficiency in myeloid or epithelial cells on the plasma levels of IL‐1β, IL‐6, and IL‐10 after DSS treatment. *Myeloid Cnlp* ^*−/−*^ mice showed increased plasma levels of IL‐1β (Figure 6A) and IL‐6 (Figure 6C) compared with *myeloid Cnlp* ^*+/+*^ mice after DSS treatment for 5 days. In contrast, *epithelial Cnlp* ^*−/−*^ mice showed reduced plasma levels of IL‐6 (Figure 6D) and IL‐10 (Figure 6F) compared with *epithelial Cnlp* ^*+/+*^ mice after DSS treatment. These data, that epithelial cell‐derived CRAMP may be associated with colon mucosal repair, prompted to us to test this possibility. Mice were given 3% DSS for 3 days, followed by normal drinking water for an additional 4 days. As shown in Figure 6G, *epithelial Cnlp* ^*−/−*^ mice showed delayed colon mucosa repair. In ulcerative foci, the re‐epithelialization was reduced in *epithelial Cnlp* ^*−/−*^ mice compared with *epithelial Cnlp* ^*+/+*^ mice. Therefore, epithelial cell‐derived CRAMP plays an important role in the repair of colon mucosal damage.

Furthermore, formylpeptide receptor 2 (*Fpr2*), a receptor for CRAMP [14], is expressed in colonic epithelial cells [20]. CRAMP expression was increased in Fpr2^+^ colon epithelial cells of the mice with colitis induced by DSS (supplementary material, Figure S6). *Fpr2* ^*−/−*^ */Cnlp* ^*−/−*^ mice showed shortened colon crypts (supplementary material, Figure S7A) and reduced numbers of Ki67^+^ epithelial cells, similar to the mice with single *Fpr2* ^*−/−*^ or *Cnlp* ^*−/−*^ gene knockout (supplementary material, Figure S7A,B), suggesting that CRAMP interacts with Fpr2 in colon epithelial cells to stimulate normal epithelial cell growth (supplementary material, Figure S8A,B). The interaction of Fpr2 in colon epithelial cells with CRAMP was also supported by accelerated healing of the epithelial cell monolayer wound by exogenous CRAMP, which was abrogated by an Fpr2 antagonist, WRW4 [33] (supplementary material, Figure S8C). CRAMP additionally induced rapid degradation of IκB‐α and phosphorylation of p38 and ERK1/2 MAP kinases in colon epithelial cells (supplementary material, Figure S8D), which were attenuated by WRW4 (supplementary material, Figure S8E). These results confirm the ability of CRAMP to stimulate epithelial cell growth through activation of the receptor Fpr2.

## Discussion

It has been reported that human LL‐37 is expressed by epithelial cells located on the luminal surface and in upper parts of colonic crypts [34]. In this study, we found that epithelial precursor cells (differentiating cells) in the crypts of naïve mice express CRAMP. In acute inflammation, CRAMP secretion into the intestinal lumen was increased, resulting in much reduced CRAMP content remaining in epithelial cells. In chronic inflammation, the production of CRAMP was also increased and CRAMP‐containing cells appeared in the differentiated region of colonic crypts. In humans, LL‐37 is likely associated with the differentiation of colon epithelial cells because *CAMP* mRNA and protein were upregulated in spontaneously differentiating Caco‐2 human colon epithelial cells as well as in HCA‐7 human colon epithelial cells treated with a differentiation‐inducing agent [34]. In *systemic Cnlp* ^*−/−*^ mice, the length of colonic crypts was significantly shortened and this was phenocopied by *epithelial Cnlp* ^*−/−*^ colon as a consequence of the reduced proliferation of epithelial cells including mucin‐producing goblet cells, due to lack of CRAMP as a cell growth and differentiation stimulant [17]. This was also evidenced by the defects in re‐epithelialization of injured colon tissues shown in *systemic Cnlp* ^*−*/−^ mice [16] and *epithelial Cnlp* ^*−/−*^ mice as shown in this study. The importance of LL‐37 in humans for inducing mucus production by colon epithelial cells was supported by observations with a human colon adenocarcinoma cell line, HT‐29, in which LL‐37 stimulates mucus synthesis through MAP kinase activation and upregulation of the transcription of *MUC* genes [27]. Thus, our results demonstrated the contribution of epithelial cell‐derived CRAMP to colon epithelial cell proliferation, goblet cell differentiation, and mucin production by using *epithelial Cnlp* ^*−/−*^ mice.

It is interesting to note that colonocytes, as absorptive cells in the colonic mucosa, have also been reported to express CRAMP [35] with yet to be clarified pathophysiological significance. In our study, unlike in epithelial cells, we did not detect CRAMP in stromal cells of mouse colon, since mature mouse stromal cells do not express CRAMP. However, human mesenchymal stem cells (MSCs) have been reported to express LL‐37 [36], which enhances the proliferation and migration of adipose‐derived stromal/stem cells (ASCs) in the colon [37]. LL‐37 secreted by human epithelium also promotes fibroblast collagen production in the lung [38]. This observation of CRAMP mediating an epithelial–stromal cell interaction merits further investigation.

It is also interesting to recognize the importance of myeloid cell‐derived CRAMP in protection of the colonic mucosa. Mounting evidence supports the protective activities of intestinal macrophages in health and disease [5, 39]. Under normal conditions, both human and mouse colons are inhabited by large numbers of bacteria, segregated from the mucosa by layers of mucus [3]. Macrophages residing in the mucosa are able to remove dying cells and also to prevent the entry and colonization of pathogens in the mucosa [5]. In inflamed gut, inflammatory macrophages are sequentially recruited to mount appropriate immune responses following an acute phase characterized by neutrophil infiltration. Ly6C^hi^ monocytes and Ly6C^int^, MHC II‐positive, CX3CR1^int^ immature macrophages accumulate at the inflamed foci and produce inflammatory cytokines including IL‐12, IL‐23, and IL‐1β which promote Th1 and Th17 cell polarization elicited by invading microorganisms that may aggravate epithelial damage [40]. Therefore, macrophages with functional deficiency may fail to protect intestinal homeostasis. CRAMP is an essential component of mouse antimicrobial defense and directly kills extracellular bacteria and inhibits the replication of intracellular bacteria [12, 41]. In mouse macrophages, endogenous CRAMP is upregulated by infection of intracellular pathogens such as *Salmonella typhimurium* [12] or *Mycobacterium smegmatis* [41]. In humans, LL‐37 is not only directly bactericidal but also serves as a mediator of vitamin D3‐induced autophagy in macrophages to activate the transcription of autophagy‐related genes *BECN1* and *Atg5*; it also participates indirectly in the elimination of intracellular bacteria [42]. PBA (4‐phenylbutyrate) promoted the co‐localization of LL‐37 with an autophagosome protein, LC3‐II, to enhance intracellular killing of *Mycobacterium tuberculosis* (Mtb) in human macrophages [43]. In our study, macrophages from *myeloid Cnlp* ^*−/−*^ mice failed to eliminate bacteria adhering to and invading colon epithelial cells, highlighting the importance of macrophage‐derived CRAMP in antimicrobial host defense. We have previously reported that CRAMP is expressed not only by macrophages but also by neutrophils [44] and dendritic cells (DCs) [14]. Neutrophils from *systemic Cnlp* ^*−/−*^ mice showed an increased level of TNF‐α after bacterial infection, but with decreased antimicrobial activity compared with wild‐type (WT) cells, indicating that CRAMP is important for normal neutrophil response to bacteria [45]. In DCs, CRAMP and its receptor Fpr2 play a non‐redundant role in cell maturation to mount an immune response [14]. Thus, myeloid cell‐derived CRAMP is important for protecting the host against bacterial invasion and for controlling immune responses.

Our previous study revealed that CRAMP plays a critical role in the maintenance of healthy microbiota in the colon, as well as in the prevention of outgrowth of certain bacteria that cause severe colitis [17]. *Cnlp* ^*−/−*^ mice housed singly showed a significantly different fecal microbiota composition compared with singly housed WT mice. However, in WT and *Cnlp* ^*−/−*^ littermates generated by mating pairs of heterozygous (*Cnlp* ^*+/−*^) parents, the composition of the fecal microbiota of the pups and heterozygous parents was similar [17]. In the present study, the pups of *myeloid Cnlp* ^*−/−*^ mice and *myeloid Cnlp* ^*+/+*^ mice or *epithelial Cnlp* ^*−/−*^ mice and *epithelial Cnlp* ^*+/+*^ mice were co‐housed to the age for the experiments that contained similar microbiota composition. These results also suggest that the phenotypes of *myeloid Cnlp* ^*−/−*^ mice or *epithelial Cnlp* ^*−/−*^ mice acquired little influence from the microbiota. However, in comparison with *myeloid Cnlp* ^*−/−*^ mice, the feces of *epithelial Cnlp* ^*−/−*^mice contained a significantly reduced abundance (*p* < 0.05) of five bacterial strains including *Murbaculaceae*, *Blautia*, *Roseburia*, *Peptococcaceae*, and *Parasutterella*, indicating that selective CRAMP deficiency in different colon cells may affect the fecal bacterial composition in the colon. However, a precise cause and effect relationship requires a more in‐depth study.

Various cytokines have been implicated in the pathologic process of IBD. Our study detected increased plasma levels of IL‐1β and IL‐6, with no changes in IL‐10 in *myeloid Cnlp* ^*−/−*^ mice compared with *myeloid Cnlp* ^*+/+*^ mice after DSS treatment for 5 days. In contrast, in *epithelial Cnlp* ^*−/−*^ mice after DSS treatment for 5 days, there was no change in the plasma levels of IL‐1β, but there was reduced IL‐6 and IL‐10 compared with *epithelial Cnlp* ^*+/+*^ mice. IL‐1β as an inflammatory cytokine was not present under homeostatic conditions [46]. On the other hand, IL‐6 and IL‐10 are linked to IBD and their reduction results in impaired healing of mucosal wounds due to decreased epithelial proliferation [32, 47, 48, 49, 50]. Therefore, it is plausible that the higher sensitivity of *myeloid Cnlp* ^*−/−*^mice to chemically induced colitis, compared with *epithelial Cnlp* ^*−/−*^ mice, may be caused by differential skewing of commensal microbiota and cytokine production.

Accumulating evidence indicates that human LL‐37 plays a crucial role in host defense against pathogen invasion, as well as demonstrating the functions of anti‐inflammation, anti‐tumorigenesis, and tissue repair in the colon [51]. The expression of *CAMP* mRNA was significantly increased in the inflamed mucosa of ulcerative colitis (UC) and Crohn's disease (CD) [52]; serum LL‐37 levels were inversely correlated with partial Mayo scores of UC patients, and the patients with high initial LL‐37 levels had a significantly better recovery than did patients with low initial LL‐37 levels after 6–18 months [53]. In the colon, LL‐37 displays an anti‐tumorigenic effect [54]. LL‐37 is strongly expressed in normal colon mucosa but is downregulated in colon cancer tissues. A comparison of the cancer cells with the adjacent noncancerous cells in the same colon revealed that in most patients, LL‐37 expression in the cancer tissue was significantly decreased [55]. LL‐37 effectively inhibits tumor growth factor‐β1‐induced EMT of colon cancer cells and proliferation of fibroblast‐supported colon cancer cells [56]. This fact was also supported by our observations in *systemic Cnlp* ^*−/−*^ mice [17] as well as in *Fpr2* ^*−/−*^ mice [20], in which a markedly increased incidence of tumors was observed in the colon with colitis. This is likely due to lack of CRAMP or its receptor Fpr2 in epithelial cells that are important for the cell turnover and mucosal repair. Therefore, the multitude of protective activities of LL‐37 in the colon, and probably other organs, defines this antimicrobial and immune‐regulatory host peptide as a promising therapeutic agent of colon diseases [57].

In summary, in dissecting the contributions of CRAMP derived from epithelial cells versus macrophages, we used genetically engineered conditional *Cnlp* ^*−/−*^ mice to clearly show the differential and distinct contribution of CRAMP derived from macrophages versus epithelial cells to colon homeostasis and inflammatory responses. Our study has also shown the collaboration of CRAMP from two cellular sources to support normal colon mucosal structure and function. However, despite these novel findings with considerable mechanistic insight, further research is warranted to explore the translational significance and potential to develop therapeutic medicine for colon diseases.

## Author contributions statement

KC designed and performed the experiments, analyzed data and drafted the manuscript. TY generated genetically engineered mice. XY, JH, AD, JM and CO assisted in experiments and data analysis. WG was responsible for mouse breeding, performed all mouse genotyping. XB and GT read and edited the manuscript. JMW supervised the study, edited the manuscript and figures. All authors approved the final manuscript.

## Supporting information


**Figure S1.** CRAMP expression in differentiating cells of the colonic crypt
**Figure S2.** CRAMP expression in colonic epithelial cells and macrophages
**Figure S3.** Detection of CRAMP in colonic mucosal macrophages and epithelial cells
**Figure S4.** Similar numbers of bacteria attached to colonic epithelium of naïve *epithelial Cnlp* ^*−/−*^ mice and *epithelial Cnlp* ^*+/+*^ mice
**Figure S5.** Reduced colon length after DSS treatment for 5 days
**Figure S6.** The expression of CRAMP in Fpr2^+^ colonic epithelial cells
**Figure S7.** Reduced epithelial cell proliferation in colonic crypts
**Figure S8.** Stimulation of colon epithelial cell proliferation by epithelium‐derived CRAMP through the receptor Fpr2

**Table S1.** Histopathological change index (HCI)
