# Supplementary material for: Distinct contributions of cathelin‐related antimicrobial peptide (CRAMP) derived from epithelial cells and macrophages to colon mucosal homeostasis
Source: J Pathol. 2021 Jan 19;253(3):339–50. doi: 10.1002/path.5572 (PMC7898386; doi:10.1002/path.5572)
Supplement: Supplementary file 1 — Figure S1. CRAMP expression in differentiating cells of the colonic crypt Figure S2. CRAMP expression in colonic epithelial cells and macrophages Figure S3. Detection of CRAMP in colonic mucosal macrophages and epithelial cells Figure S4. Similar numbers of bacteria attached to colonic epithelium of naïve epithelial Cnlp −/− mice and epithelial Cnlp +/+ mice Figure S5. Reduced colon length after DSS treatment for 5 days Figure S6. The expression of CRAMP in Fpr2+ colonic epithelial cells Figure S7. Reduced epithelial cell proliferation in colonic crypts Figure S8. Stimulation of colon epithelial cell proliferation by epithelium‐derived CRAMP through the receptor Fpr2 [file PATH-253-339-s001.docx]

**Distinct contributions of cathelin-related antimicrobial peptide (CRAMP) derived from epithelial cells and macrophages to colon mucosal homeostasis**

K Chen *et al. J Pathol* DOI: 10.1002/path.5572

**Supplementary Figures S1–S8**

**
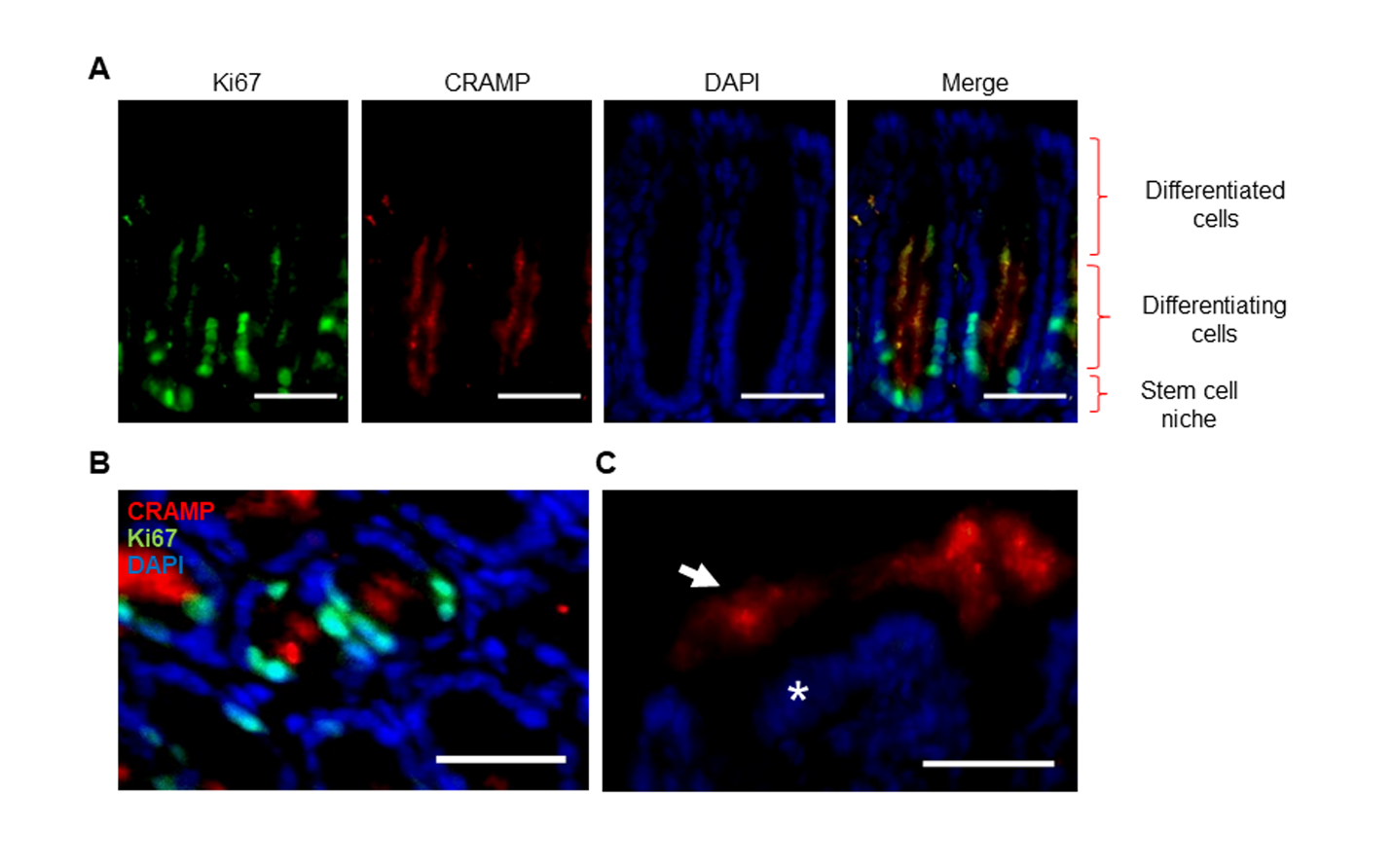
**

**Figure S1. CRAMP expression in differentiating cells of the colonic crypt.**

(A) CRAMP expression in Ki67^+^ cells of the colonic crypt. Green: Ki67; red: CRAMP; blue: DAPI. Scale bar = 30 μm. (B) Oblique section through the differentiating region of colonic crypts. Green: Ki67; red: CRAMP; blue: DAPI. Scale bar = 20 μm. (C) Surface of the colonic mucosa. Red: CRAMP; blue: DAPI. Arrow: CRAMP is located on the surface of the colonic mucosa. Asterisk: colon mucosa. Scale bar = 20 μm.


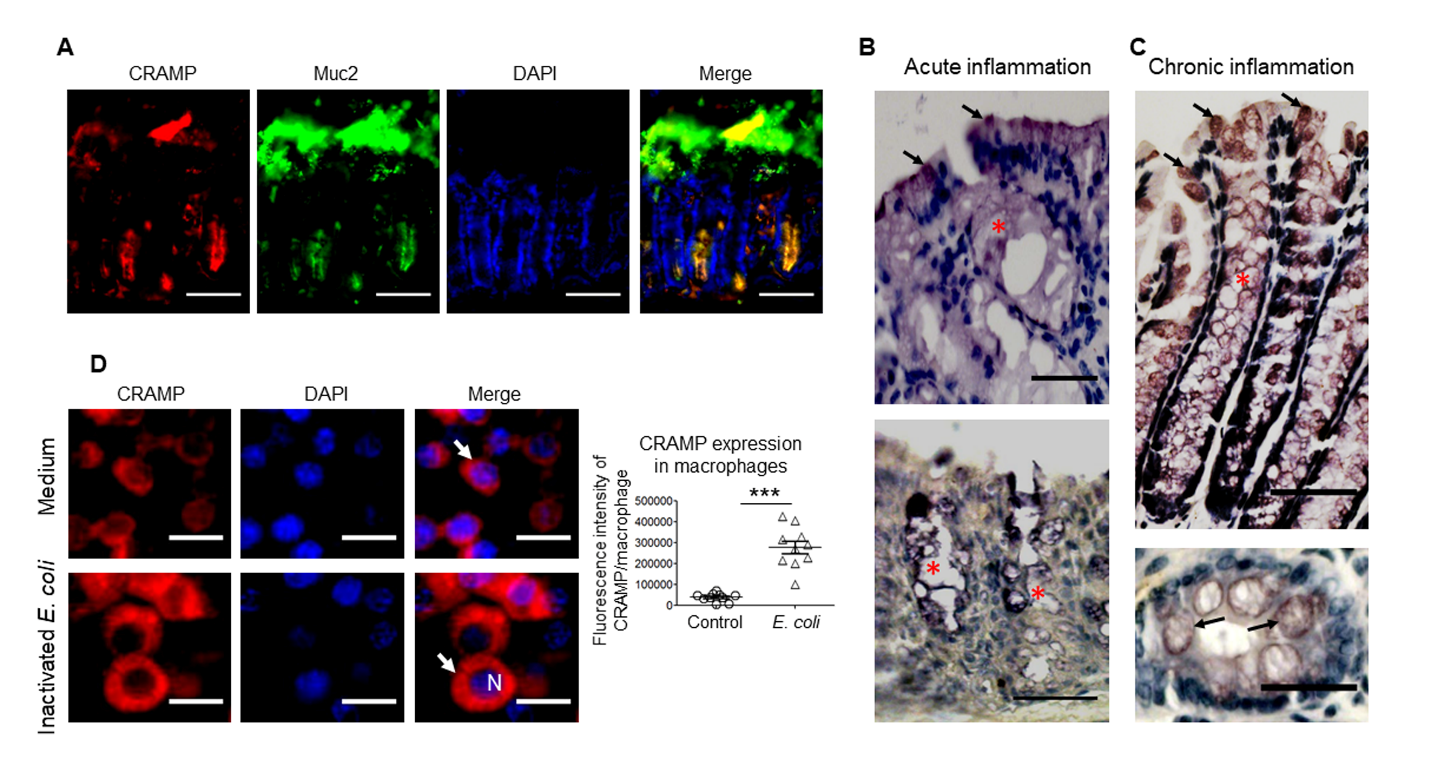


**Figure S2. CRAMP expression in colonic epithelial cells and macrophages.**

(A) CRAMP co-located with Muc2. Red: CRAMP; green: Muc2; blue: DAPI. Scale bar = 50 μm. (B) CRAMP secretion into the intestinal lumen was increased, resulting in a much reduced CRAMP content remaining in epithelial cells in acute inflammation. Asterisk: colonic crypt; arrow: CRAMP^+^ cells in a differentiated area of a colonic crypt. Brown color: CRAMP^+^ cells. Scale bar = 30 μm for upper panel and 20 μm for lower panel. (C) CRAMP^+^ cells expanded into the differentiated area of colonic crypts under chronic inflammation. Upper panel: longitudinal section through colonic crypts. Asterisk: colonic crypt; arrow: CRAMP^+^ cells in a differentiated area of a colonic crypt. Brown color: CRAMP^+^ cells. Scale bar = 40 μm. Lower panel: oblique section of a crypt. Arrow: CRAMP^+^ cells. Scale bar = 15 μm. (D) Detection of increased CRAMP expression by macrophages stimulated by inactivated *E. coli*. Red: CRAMP; blue: DAPI. Arrow: macrophages; N: nuclei. Scale bar = 10 µm (left panels). Right panel: quantitation of CRAMP-positive staining spots per macrophage. The immunofluorescence intensity per macrophage is shown. *n* = 10 fields per group. ****p* < 0.001.


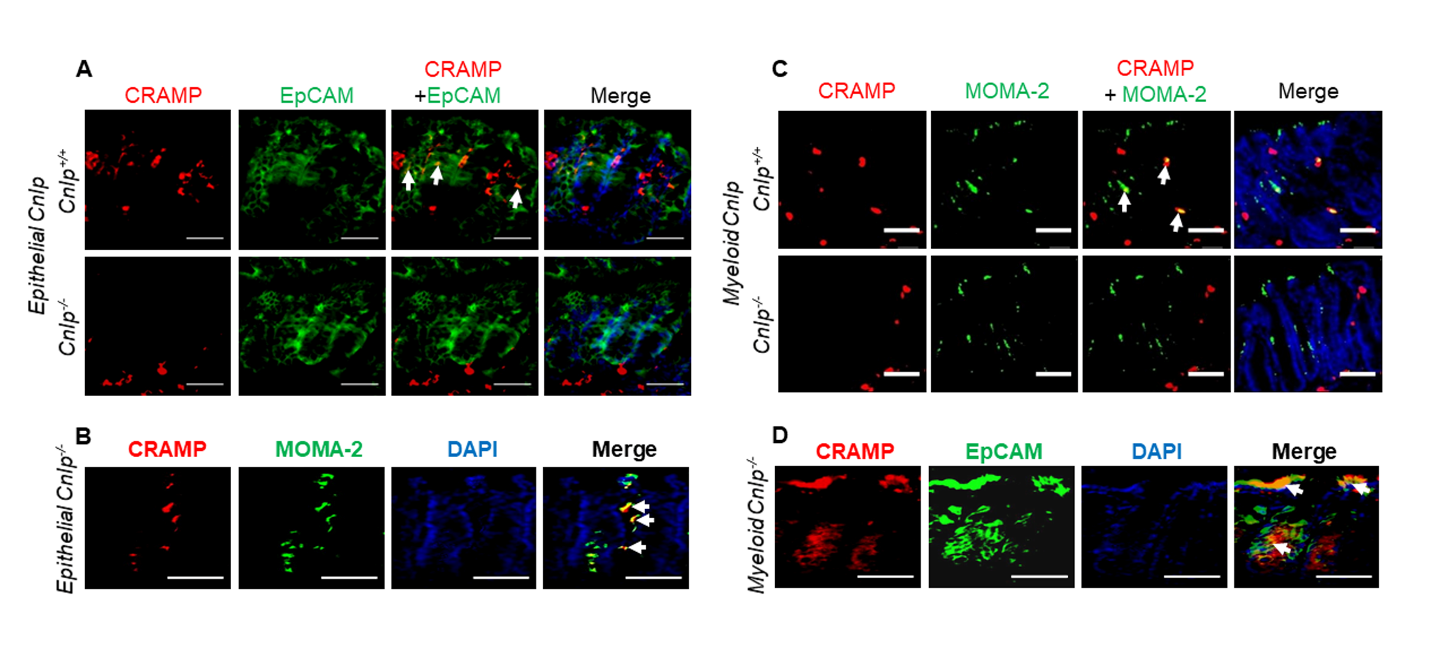


**Figure S3. Detection of CRAMP in colonic mucosal macrophages and epithelial cells.**

(A) Upper panel: CRAMP was detected in colonic epithelial cells of *epithelial Cnlp^+/+^* mice. Lower panel: no CRAMP was detected in colonic epithelial cells of *epithelial Cnlp^−/−^* mice. Red: CRAMP; green: EpCAM^+^ epithelial cells; blue: DAPI. White arrows: EpCAM^+^ epithelial cells containing CRAMP. Scale bar = 30 μm. (B) Representative images showing CRAMP^+^ and MOMA-2^+^ macrophages in *epithelial Cnlp^−/−^* mouse colon (white arrows). Red: CRAMP; green: MOMA-2; blue: DAPI. Scale bar = 30 μm. (C) Upper panel: CRAMP^+^ and MOMA-2^+^ macrophages in the colonic mucosa of *myeloid Cnlp^+/+^*mice. Lower panel: no CRAMP was detected in macrophages in the colonic mucosa of *myeloid Cnlp^−^**^/−^* mice. Red: CRAMP; green: MOMA-2; blue: DAPI. White arrows: CRAMP^+^ and MOMA-2^+^ macrophages. Scale bar = 30 μm. (D) Representative images showing CRAMP^+^ and EpCAM^+^ epithelial cells in *myeloid Cnlp^−/−^* mouse colon (white arrows). Red: CRAMP; green: EpCAM^+^ epithelial cells; blue: DAPI. Scale bar = 30 μm.

**Figure S4. Similar numbers of bacteria attached to colonic epithelium of naïve *epithelial Cnlp****^−/−^* **mice and *epithelial Cnlp^+/+^* mice.**

(A) Epithelial cell-derived CRAMP deficiency does not alter the microbiota composition in the colon of *epithelial Cnlp^−/−^* mice co-housed with *epithelial Cnlp^+/+^* mice. Fecal bacterial DNA was isolated from four *epithelial Cnlp^+/+^* mice and four *epithelial* *Cnlp^−/−^* mice at the age of 12 weeks. 16S sequence frequencies were analyzed by 16S amplicon high-throughput sequencing of fecal microbiota. Data are shown as a heat map of classified sequences. (B) The number of bacteria attached to colon mucosa of *epithelial Cnlp^−/−^* mice was not increased. Upper panels: representative images are shown. Scale bar = 30 μm. Lower panel: Counts of attached bacteria in the colon mucosa of *epithelial Cnlp^−/−^* mice and *epithelial Cnlp ^+/+^* mice. *n* = 28 fields per group; eight mice per group. ns = no significant difference.


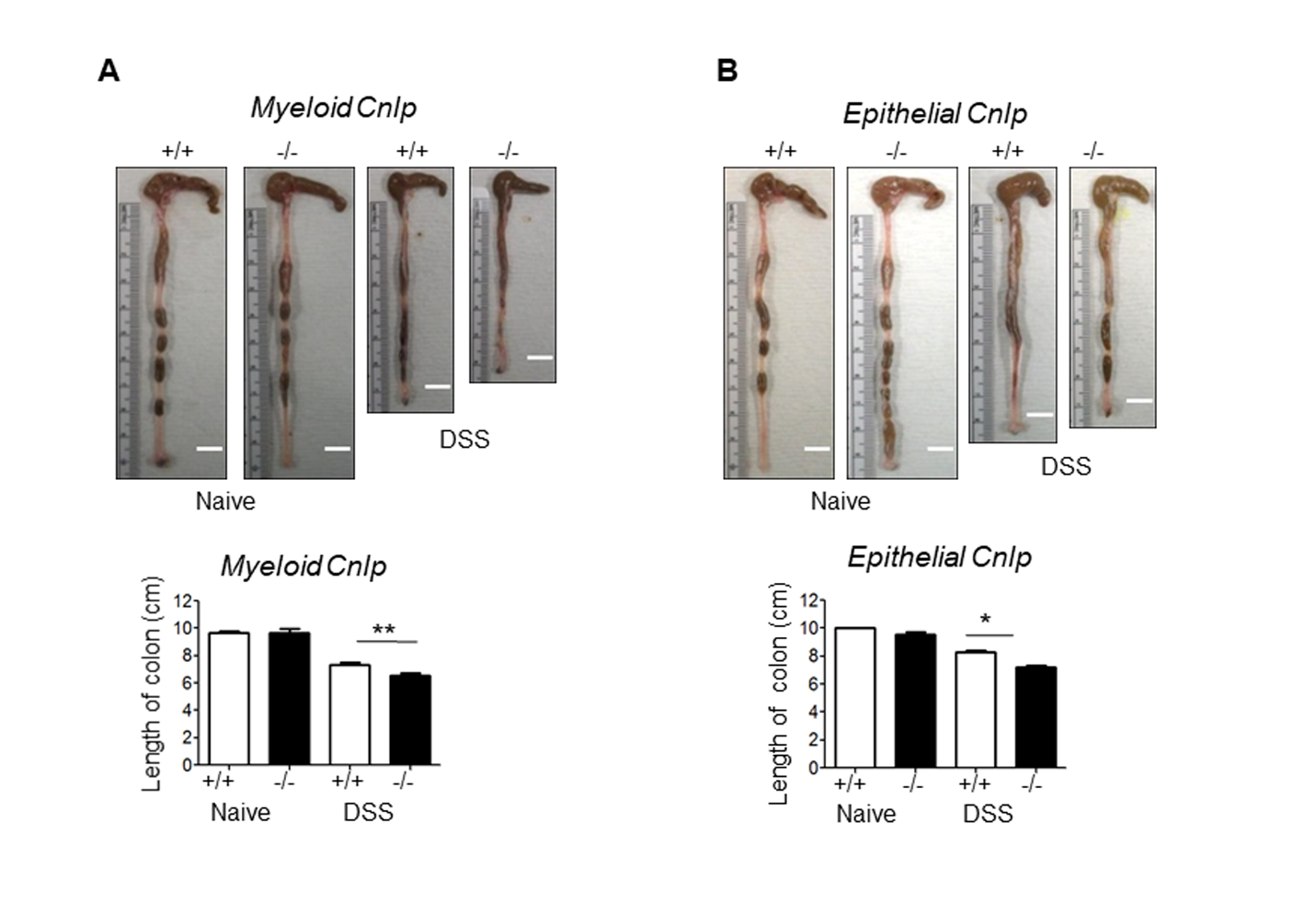


**Figure S5. Reduced colon length after DSS treatment for 5 days.**

(A) Reduced colon length of *myeloid Cnlp^−/−^* mice at 5 days after 3% DSS treatment. Upper panels: representative images are shown. Scale bar = 1 cm. Lower panel: colon length in *myeloid Cnlp^−/−^* and *myeloid Cnlp^+/+^* mice after DSS treatment for 5 days. *n* = 10–12 per group. ***p* < 0.01. (B) Reduced colon length of *epithelial Cnlp^−/−^* mice at 5 days after 3% DSS treatment. Upper panels: representative images are shown. Lower panel: the length of colons in *epithelial Cnlp^−/−^* and *epithelial Cnlp^+/+^* mice after DSS treatment for 5 days. *n* = 10–12 per group. **p* < 0.05.


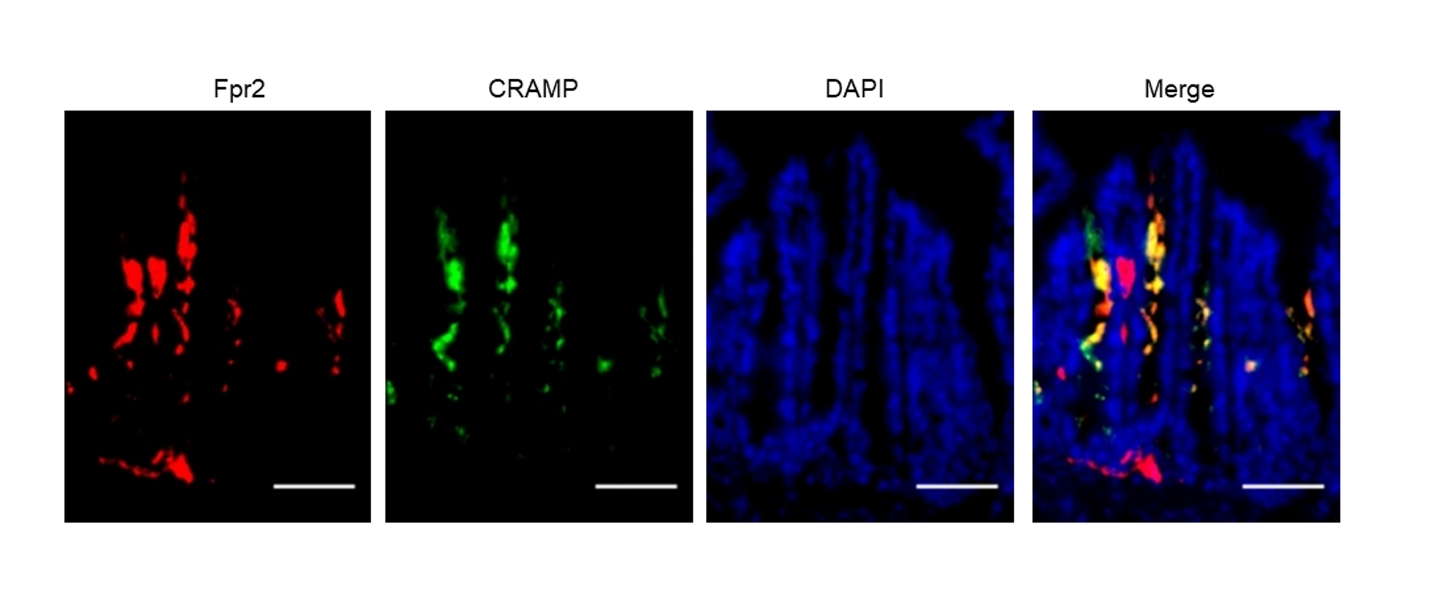


**Figure S6. The expression of CRAMP in Fpr2^+^ colonic epithelial cells.**

CRAMP expression in Fpr2^+^ colon epithelial cells of mice. *Myeloid* *Cnlp^−/−^* mice (colon epithelial cells express CRAMP) were treated with 3% DSS for 5 days and the colon mucosa was stained with antibodies to detect CRAMP and Fpr2. DAPI was used to stain nuclei. Red: Fpr2; green: CRAMP; blue: DAPI. Scale bar = 50 μm.


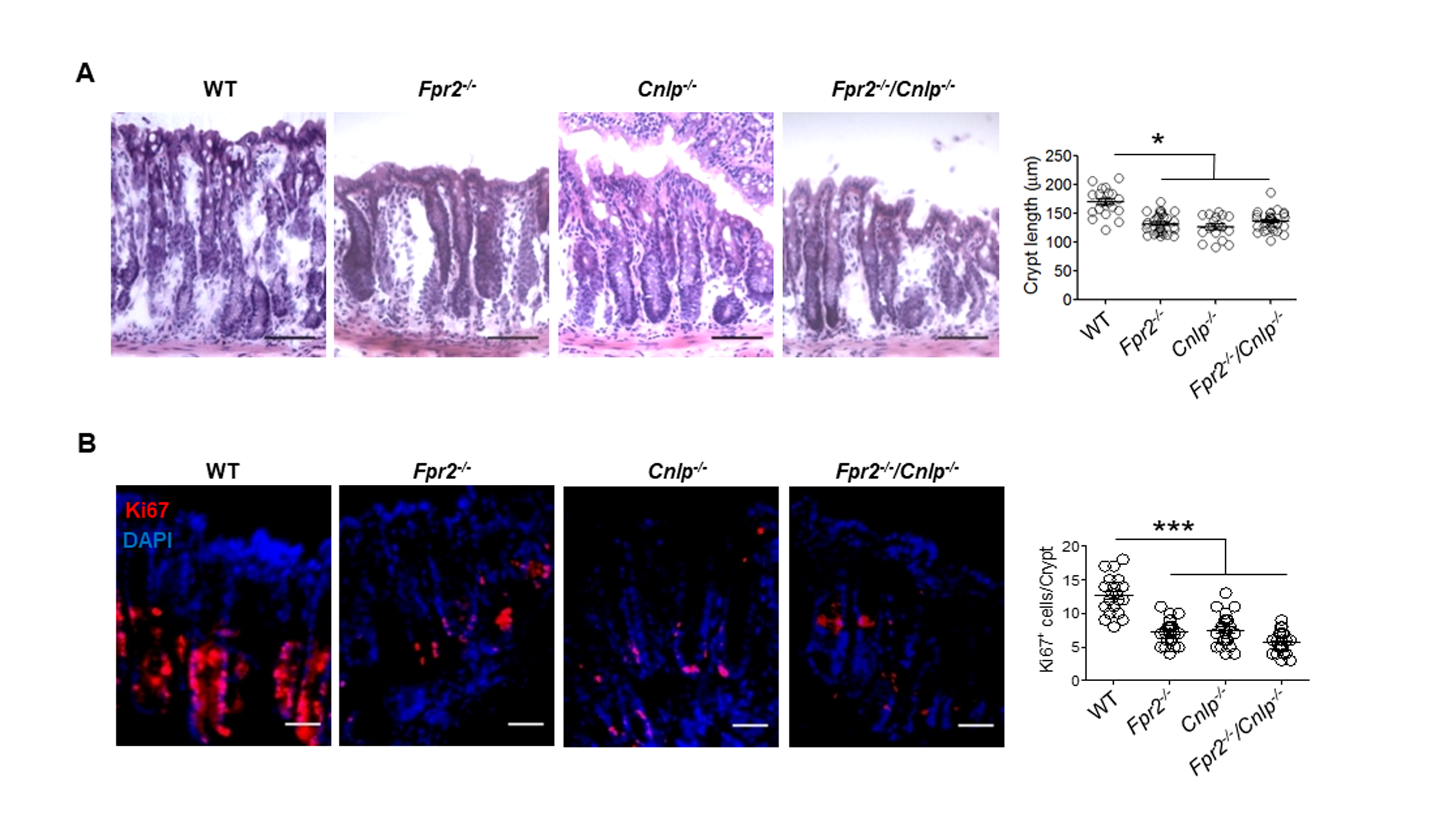


**Figure S7. Reduced epithelial cell proliferation in colonic crypts.**

(A) Colonic crypts in the *systemic Fpr2^−/−^*, *systemic Cnlp^−/−^*, and *Fpr2/Cnlp double* (*dl*) *^−/−^* mice are shorter than those in WT mice. Left panels: representative images are shown. H&E staining. Scale bar = 50 μm. Right panel: quantitative analysis of crypt lengths. *n* = 10–14 per group. **p*< 0.05. (B) Fewer Ki67^+^ cells were detected in the colonic crypts of *systemic Fpr2^−/−^*, *systemic Cnlp^−/−^*, and *Fpr2/Cnlp* *double* (*dl*) *^−/−^* mice than in those of WT mice. Left panel: representative images are shown. Red: Ki67; blue: DAPI. Scale bar =  30 μm. Right panel: the number of Ki67^+^ cells per crypt. *n* = 10–14 per group. ****p* < 0.001.


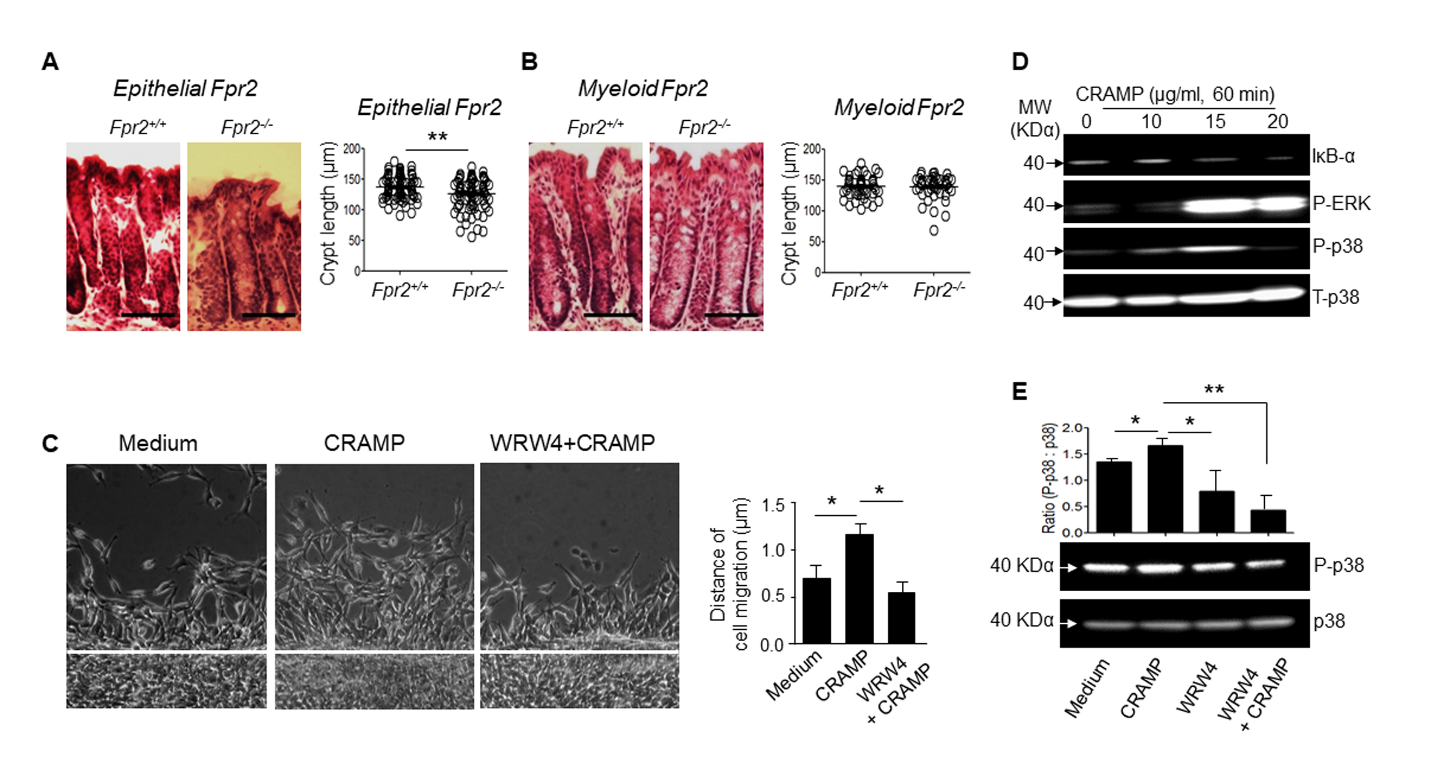


**Figure S8. Stimulation of colon epithelial cell proliferation by epithelium-derived CRAMP through the receptor Fpr2.**

(A) Colonic crypts in *epithelial Fpr2^−/−^* mice are shorter. Colon sections from naïve *epithelial Fpr2^−/−^* mice and *epithelial Fpr2^+/+^* mice were stained with H&E for measurement of crypt length. Left panels: representative images are shown. Scale bar = 30 μm. Right panel: quantitative analysis of colon crypt lengths in *epithelial Fpr2^−/−^* mice and *epithelial Fpr2^+/+^* mice. *n* = 18 per group. ***p* < 0.01. (B) Similar crypt lengths in *myeloid Fpr2^−/−^* mice and *myeloid Fpr2^+/+^* mice. Left panel: representative images are shown. H&E staining. Scale bar = 50 μm. Right panel: quantitative analysis of colon crypt length. *n* = 18 per group. (C) Blockage of CRAMP-promoted CT26 epithelial cell wound closure by the Fpr2 antagonist WRW4 (20 μg/ml). Left panels: representative images are shown. Right panel: distance migrated by CT26 cells toward the centerline of the monolayer wounds. **p* < 0.05. (D) Activation of IκB and phospho (P)-ERK1/2 and P-p38 in CT26 cells by exogeneous CRAMP. CT26 cells were cultured in the absence or presence of CRAMP at the indicated concentrations at 37 °C for 60 min. The cell lysates were analyzed for total IκB, P-ERK1/2, and P-p38. Total p38 was used as a loading control. (E) Attenuation of CRAMP-induced P-p38 by the Fpr2 antagonist WRW4. CT26 cells were pretreated with WRW4 (10 μg/ml) for 1 h, followed by CRAMP (10 μg/ml) for an additional 1 h. The cell lysates were analyzed for P-p38. Total p38 was used as a loading control. Upper panel: ratio of P-p38:p38 analyzed by densitometry. Lower panel: representative images of western blotting are shown. **p* < 0.05, ***p* < 0.01.
