# Supplementary material for: Distinct contributions of cathelin‐related antimicrobial peptide (CRAMP) derived from epithelial cells and macrophages to colon mucosal homeostasis
Source: J Pathol. 2021 Jan 19;253(3):339–50. doi: 10.1002/path.5572 (PMC7898386; doi:10.1002/path.5572)
Supplement: Supplementary file 2 — Table S1. Histopathological change index (HCI) [file PATH-253-339-s002.docx]

**Distinct contributions of cathelin-related antimicrobial peptide (CRAMP) derived from epithelial cells and macrophages to colon mucosal homeostasis**

K Chen *et al. J Pathol* DOI: 10.1002/path.5572

**Supplementary Table S1**

**Table S1.** Histopathological change index (HCI)

| **Score** | **Crypt damage** | **Inflammatory cell (iCell) infiltration** | **Extent of crypt damage and inflammation** |
| --- | --- | --- | --- |
| **0** | Intact | Few iCells | Normal |
| **1** | Loss of the basal 1/3 of crypt | 1–3 iCells per field in the submucosa | 1/5 colon |
| **2** | Loss of basal 1/2 of the crypt | >3 iCells per field in the submucosa | 2/5 colon |
| **3** | Loss of basal 2/3 of the crypt | 1–3 iCells per field in the mucosa | 3/5 colon |
| **4** | Entire loss of the crypt | >3 iCells per field in the mucosa | 4/5 colon |
| **5** | Loss of crypt and surface epithelia | Numerous iCells in the epithelium | Whole colon |
